# Supplementary material for: Gi/o protein-coupled receptor inhibition of beta-cell electrical excitability and insulin secretion depends on Na+/K+ ATPase activation
Source: Nat Commun. 2022 Oct 29;13:6461. doi: 10.1038/s41467-022-34166-z (PMC9617941; doi:10.1038/s41467-022-34166-z)
Supplement: Supplementary file 1 — Supplementary Information [file 41467_2022_34166_MOESM1_ESM.pdf]

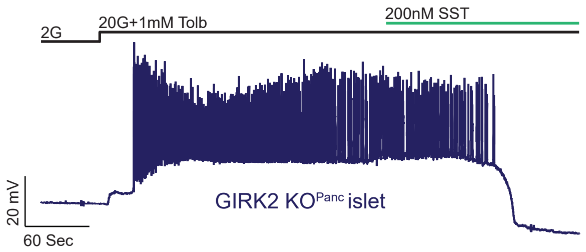

Supplementary Fig. 1: SST stimulates V_m hyperpolarization of GIRK2KO β-cells. Representative GIRK2 KO^{Panc} β-cell V_m recording illustrating a typical SST response.

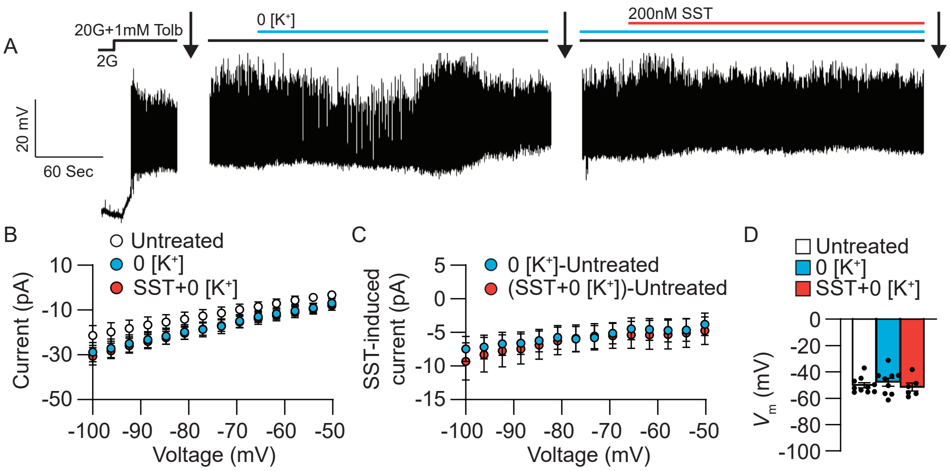

Supplementary Fig. 2: Extracellular K⁺ is required for activation of SST-induced β-cell currents. (A) Representative WT β-cell V_m recording showing a typical SST response in the absence of extracellular K⁺ (0 [K⁺]). Whole-cell β-cell currents were measured (indicated by arrows) in response to a voltage ramp protocol (see Fig. 1A inset). (B) WT β-cell currents before treatment (white), after 0 [K⁺] (light blue), and after 0 [K⁺]+SST (orange; *n*=10). (C) 0 [K⁺]-induced (blue) and 0 [K⁺]+SST-induced (orange) WT β-cell currents (*n*=10). (D) WT β-cell V_m before treatment white; *n*=10), after 0 [K⁺] (blue; *n*=9), and after 0 [K⁺]+SST (orange; *n*=6). Statistical analysis was conducted using a paired two-sided two-sample t-test (C) or one-way ANOVA with Šidák's post-hoc multiple comparisons tests (B and D). Source data and exact P values are provided as a Source Data file.

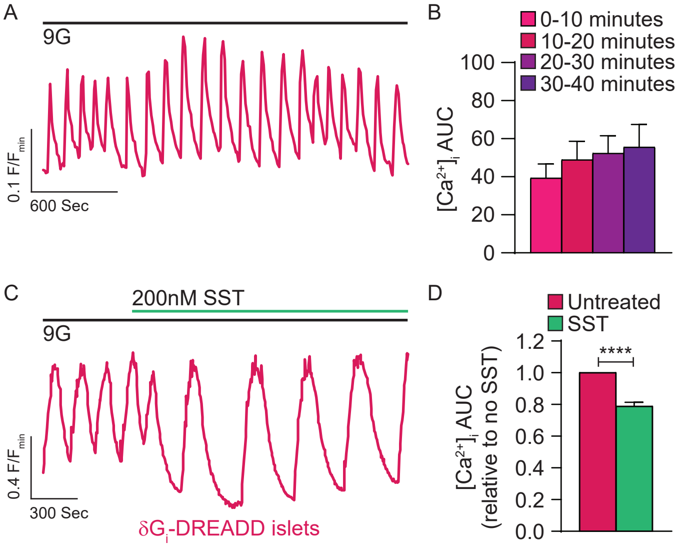

Supplementary Fig. 3: SST decreases islet $[Ca^{2+}]_i$ in the presence of 9mM glucose. (A) Representative $\delta G_{i/o}$-DREADD islet Ca^{2+} oscillations (F/F_{min}) at 9mM glucose (9G). (B) $\delta G_{i/o}$-DREADD islet $[Ca^{2+}]_i$ AUC as a function of time (*n*=14). (C) Representative $\delta G_{i/o}$-DREADD islet SST $[Ca^{2+}]_i$ response at 9G. (D) $\delta G_{i/o}$-DREADD islet $[Ca^{2+}]_i$ AUC (sum of 10 minutes; relative to before SST) before (dark pink) and after SST (green; *n*=48). Statistical analysis was conducted using a one-sample t-test (D) or one-way ANOVA with Šidák's post-hoc multiple comparisons tests (B); ****P<0.0001. Source data and exact P values are provided as a Source Data file.

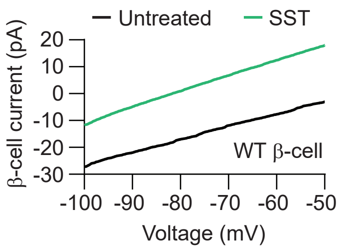

Supplementary Fig. 4: SST induces outward β-cell currents. Representative WT β-cell current in the presence of 20mM glucose and 1mM tolbutamide (black) and after addition of 200nM SST (green).

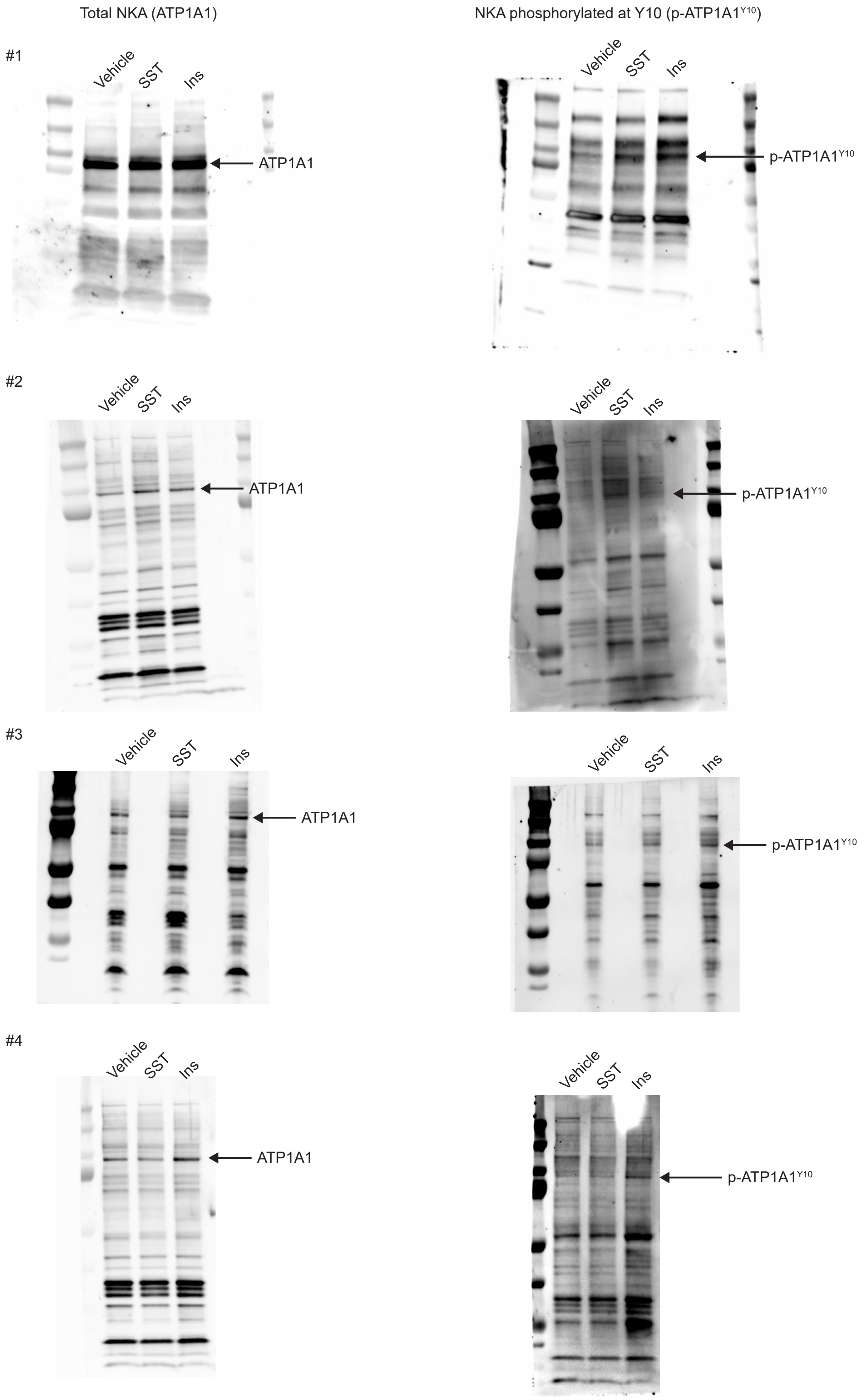

Supplementary Fig. 5: Uncropped and unprocessed immunoblot scans
