# Supplementary material for: Gi/o protein-coupled receptor inhibition of beta-cell electrical excitability and insulin secretion depends on Na+/K+ ATPase activation
Source: Nat Commun. 2022 Oct 29;13:6461. doi: 10.1038/s41467-022-34166-z (PMC9617941; doi:10.1038/s41467-022-34166-z)
Supplement: Supplementary file 3 — Reporting Summary [file 41467_2022_34166_MOESM3_ESM.pdf]

Corresponding author(s): David A. Jacobson
Last updated by author(s): Jul 22, 2022

Reporting Summary

Nature Portfolio wishes to improve the reproducibility of the work that we publish. This form provides structure for consistency and transparency in reporting. For further information on Nature Portfolio policies, see our Editorial Policies and the Editorial Policy Checklist.

Statistics

For all statistical analyses, confirm that the following items are present in the figure legend, table legend, main text, or Methods section.

n/a	Confirmed	
☐	☒	The exact sample size (*n*) for each experimental group/condition, given as a discrete number and unit of measurement
☐	☒	A statement on whether measurements were taken from distinct samples or whether the same sample was measured repeatedly
☐	☒	The statistical test(s) used AND whether they are one- or two-sided<br>*Only common tests should be described solely by name; describe more complex techniques in the Methods section.*
☐	☒	A description of all covariates tested
☐	☒	A description of any assumptions or corrections, such as tests of normality and adjustment for multiple comparisons
☐	☒	A full description of the statistical parameters including central tendency (e.g. means) or other basic estimates (e.g. regression coefficient) AND variation (e.g. standard deviation) or associated estimates of uncertainty (e.g. confidence intervals)
☐	☒	For null hypothesis testing, the test statistic (e.g. *F*, *t*, *r*) with confidence intervals, effect sizes, degrees of freedom and *P* value noted<br>*Give P values as exact values whenever suitable.*
☒	☐	For Bayesian analysis, information on the choice of priors and Markov chain Monte Carlo settings
☒	☐	For hierarchical and complex designs, identification of the appropriate level for tests and full reporting of outcomes
☒	☐	Estimates of effect sizes (e.g. Cohen's *d*, Pearson's *r*), indicating how they were calculated

Our web collection on statistics for biologists contains articles on many of the points above.

Software and code

Policy information about availability of computer code

Data collection	Zeiss Zen 2.3, Nikon Elements 5.11.03, Bio-Rad Image Lab 5.0, and Axon pCLAMP10 10.7 were used to collect experimental data.
Data analysis	Axon Clampfit 11.2, Bio-Rad Image Lab 5.0, Microsoft Excel 2019, GraphPad Prism 9.2.0, ImageJ Fiji image processing pack 1.53t 24 August 2022, MATLAB R2019b were utilized for data analysis.

For manuscripts utilizing custom algorithms or software that are central to the research but not yet described in published literature, software must be made available to editors and reviewers. We strongly encourage code deposition in a community repository (e.g. GitHub). See the Nature Portfolio guidelines for submitting code & software for further information.

Data

Policy information about availability of data

All manuscripts must include a data availability statement. This statement should provide the following information, where applicable:

- Accession codes, unique identifiers, or web links for publicly available datasets
- A description of any restrictions on data availability
- For clinical datasets or third party data, please ensure that the statement adheres to our policy

The datasets generated during and/or analyzed during the current study are available as a Source Data File.

Human research participants

Policy information about studies involving human research participants and Sex and Gender in Research.

Reporting on sex and gender	This work did not include human research participants. All human islet donors were deidentified prior to reception by our lab. The sex of all human islet donors is provided in Table 1. Because the number of donors was low, sex-based data analysis was not possible.
Population characteristics	This work did not include human research participants; however, covariate relevant population characteristics for all human islet donors (e.g. age, race, body mass index) are provided in Table 1. The number of donors was insufficient to analyze these effects.
Recruitment	Human research participants were not recruited for this work.
Ethics oversight	All studies detailed here were approved by the Vanderbilt University Health Sciences Committee Institutional Review Board (IRB# 110164).

Note that full information on the approval of the study protocol must also be provided in the manuscript.

Field-specific reporting

Please select the one below that is the best fit for your research. If you are not sure, read the appropriate sections before making your selection.

☒ Life sciences ☐ Behavioural & social sciences ☐ Ecological, evolutionary & environmental sciences

For a reference copy of the document with all sections, see nature.com/documents/nr-reporting-summary-flat.pdf

Life sciences study design

All studies must disclose on these points even when the disclosure is negative.

Sample size	Sample sizes were determined utilizing power analysis to allow rejection of the null hypothesis.
Data exclusions	No data was excluded from analysis.
Replication	In all cases, results are the average of at least 3 independent biological replicates to ensure reproducibility. All attempts at replication were successful.
Randomization	All samples were randomly allocated into experimental groups. Covariates were controlled for by ensuring experimental conditions were identical for all biological replicates.
Blinding	Assay technicians were blinded to group allocation during data collection.

Reporting for specific materials, systems and methods

We require information from authors about some types of materials, experimental systems and methods used in many studies. Here, indicate whether each material, system or method listed is relevant to your study. If you are not sure if a list item applies to your research, read the appropriate section before selecting a response.

Materials & experimental systems

n/a	Involved in the study	
☐	☒	Antibodies
☒	☐	Eukaryotic cell lines
☒	☐	Palaeontology and archaeology
☐	☒	Animals and other organisms
☒	☐	Clinical data
☒	☐	Dual use research of concern

Methods

n/a	Involved in the study	
☒	☐	ChIP-seq
☒	☐	Flow cytometry
☒	☐	MRI-based neuroimaging

Antibodies

Antibodies used	- Mouse anti-ATP1A1 (catalog #: MA3-928; Thermo Fisher)

Antibodies used

- Guinea pig anti-insulin (catalog #: 20-IP35; Fitzgerald, North Acton, MA)
- Donkey anti-mouse Alexa Fluor 647 (catalog #: 715-606-150; Jackson ImmunoResearch, West Grove, PA)
- Donkey anti-guinea pig Alexa Fluor 488 (catalog #: 706-546-148; Jackson ImmunoResearch)
- Rabbit anti-phospho-ATP1A1 (Y10) (catalog #: PA5-17061; Thermo Fisher)
- Goat anti-rabbit HRP-conjugated secondary (catalog #: W4011; Promega, Madison, WI), and goat anti-mouse HRP-conjugated secondary (catalog #: W4021; Promega)

Validation

- Mouse anti-ATP1A1 (catalog #: MA3-928; Thermo Fisher); this antibody was verified on tissue with ATP1A1 knockdown to ensure that the antibody binds to the antigen stated.
- Guinea pig anti-insulin (catalog #: 20-IP35; Fitzgerald, North Acton, MA); this antibody was verified by immunofluorescence staining of mouse and human pancreatic secretions.
- Donkey anti-mouse Alexa Fluor 647 (catalog #: 715-606-150; Jackson ImmunoResearch, West Grove, PA); Based on immunoelectrophoresis and/or ELISA, the antibody reacts with whole molecule mouse IgG. It also reacts with the light chains of other mouse immunoglobulins. No antibody was detected against non-immunoglobulin serum proteins.
- Donkey anti-guinea pig Alexa Fluor 488 (catalog #: 706-546-148; Jackson ImmunoResearch); Based on immunoelectrophoresis and/or ELISA, the antibody reacts with whole molecule guinea pig IgG. It also reacts with the light chains of other guinea pig immunoglobulins. No antibody was detected against non-immunoglobulin serum proteins.
- Rabbit anti-phospho-ATP1A1 (Y10) (catalog #: PA5-17061; Thermo Fisher); this antibody was verified by immunoblot to ensure that the antibody binds to the antigen stated.
- Goat anti-rabbit HRP-conjugated secondary (catalog #: W4011; Promega, Madison, WI), and goat anti-mouse HRP-conjugated secondary (catalog #: W4021; Promega); extensively used in the literature

References

Hu, D. et al. (2012) Novel insight into KLF4 proteolytic regulation in estrogen receptor signaling and breast carcinogenesis. J. Biol. Chem. 287, 13584–97.
Hesse, E. et al. (2010) Zfp521 controls bone mass by HDAC3-dependent attenuation of Runx2 activity. J. Cell Biol. 191, 1271–83.
Massaad, C.A., Washington, T.M., Pautler, R.G. and Klann, E. (2009) Overexpression of SOD-2 reduces hippocampal superoxide and prevents memory deficits in a mouse model of Alzheimer's disease. Proc. Natl. Acad. Sci. USA 106, 13576–81.
Berkowitz, O., Jost, R., Pollmann, S. and Masle, J. (2008) Characterization of TCTP, the translationally controlled tumor protein, from Arabidopsis thaliana. Plant Cell 20, 3430–47.
Robertson, J.B., Stowers, C.C., Boczko, E. and Johnson, C.H. (2008) Real-time luminescence monitoring of cell-cycle and respiratory oscillations in yeast. Proc. Natl. Acad. Sci. USA 105, 17988–93.

Animals and other research organisms

Policy information about studies involving animals; ARRIVE guidelines recommended for reporting animal research, and Sex and Gender in Research

Laboratory animals

10- to 16-week old, age-matched males on a C57Bl6/J background (Stock #: 000664; The Jackson Laboratory (JAX), Bar Harbor, ME). 10- to 16-week old, age-matched males expressing aB6.129-Gt(ROSA)26Sortm1(CAG-CHRM4*,-mCitrine)Ute/J (Stock #: 026219; JAX) and an SST-IRES-Cre (Stock #: 013044; JAX). All animals were housed in a Vanderbilt University IACUC (protocol # M1600063-01) approved facility on a 12-hour light/dark cycle with access to standard chow (Lab Diets, 5L0D) ad libitum. Room conditions were maintained between 65-75°F (~18-23°C) with 40-60% humidity.

Wild animals

This study did not involve wild animals.

Reporting on sex

Human islets derived from male and female donors as well as mouse islets from male animals were used in this study.

Field-collected samples

This study did not involve the collection of field samples.

Ethics oversight

All mouse studies were approved by the Vanderbilt University IACUC (protocol # M1600063-01).

Note that full information on the approval of the study protocol must also be provided in the manuscript.
